# Introduction to the EJPH supplement ‘E-mental health: exploring the evidence base and stakeholders’ perspectives on Internet-based interventions for the prevention of mental health conditions’

**DOI:** 10.1093/eurpub/ckz087

**Published:** 2021-07-07

**Authors:** Corinna Jacobi, Karin Waldherr, Lisa M Klesges, Thomas Ernst Dorner, C Barr Taylor

**Affiliations:** 1 Technische Universität Dresden, Faculty of Psychology, Chair of Clinical Psychology and E-Mental Health, Dresden, Germany; 2 Ferdinand Porsche FernFH—Distance Learning University of Applied Sciences, Wiener Neustadt, Austria; 3 School of Public Health, University of Memphis, Memphis, TN, USA; 4 Department of Social and Preventive Medicine, Medical University of Vienna, Centre for Public Health, Vienna, Austria; 5 Center for m^2^Health, Palo Alto University, Palo Alto, CA, USA; 6 Department of Psychiatry & Behavioral Sciences, Stanford University, Palo Alto, CA, USA

## Background

Mental illness represents an enormous personal, social and societal burden for European citizens^1^ calling for the need to expand existing models of mental healthcare delivery. In Europe, the Internet is a key source of health information,^2^ and technology-enhanced (psychological) interventions such as Internet- and mobile-delivered applications (‘eHealth’^3^ and ‘m-Health’^4^) have become increasingly popular and studied. There is already strong evidence of the efficacy of online interventions for the prevention and treatment of several psychological disorders^5^^,^^6^ and meta-analyses show effect sizes similar to face-to-face interventions.^7^

In 2015, a large collaborative network, ‘Integrating technology into mental healthcare delivery in Europe (ICare)’, funded by the European Commission as part of the Horizon 2020 programme started in six European countries (Germany, Austria, Switzerland, Great Britain, The Netherlands, Spain). The overall aim of ICare was to establish a novel, comprehensive and effective model of mental health service delivery. ICare encompasses mental health promotion, risk detection, disease and relapse prevention of common mental health disorders (CMHD) and guided self-help for eating disorder (ED) patients as well as caregivers of people with an ED delivered through a common online platform.^8^ The design of ICare involved four different phases:^8^ (i) adoption by relevant settings, (ii) screening and recruitment of participants, (iii) active intervention delivery and (iv) synthesis of results. The aim was to translate the ICare interventions into real-world contexts (healthcare systems, schools, universities). Thus, knowledge about adoption and implementation in different settings is an important prerequisite for a successful broad-scale dissemination.^9^ Therefore, in the first phase of ICare, a stakeholder survey was carried out in each participating country in the settings, in which ICare interventions were implemented.

## Scope and content of the supplement

This supplement consists of two parts. The first part summarizes current research on Internet-based treatment and prevention for some of the most CMHD. The second part of the supplement is dedicated to the ICare Stakeholder Survey.

Part I—Evidence: As an introduction to the topic, Taylor et al. present an overview of existing systematic reviews and meta-analyses on Internet-based interventions for the treatment of depression, anxiety, EDs and substance abuse. The authors identified important research gaps including the need for more evidence on how to increase engagement, on more tailored interventions for various populations and on interventions delivered by mobile phones. The next article by Buntrock et al. summarizes the evidence on economic evaluations of Internet- and mobile-based interventions for substance use disorders. The results regarding cost-effectiveness of these interventions are promising. However, the authors emphasize that more economic evaluations are needed. The article of Díaz-García et al. presents a systematic review on the theoretical adequacy, methodological quality and efficacy of Internet-based interventions to promote well-being and resilience. They found a tendency for larger effects of programmes with a clear assessment theory, which underlines the need of strong theoretical foundation of Internet-based interventions. Finally, Zeiler et al. and Nacke et al. present systematic reviews evaluating reach, adoption, implementation and maintenance of Internet-based interventions to prevent EDs in adolescents and adults, respectively. Both reviews revealed a lack of reporting of external validity indicators. Overall, the results of the articles in the first part of this supplement show that Internet-based interventions are feasible and effective adjuncts to face-to-face interventions in order to deal with the increased need for mental health promotion and treatment. However, they identified also a range of research gaps, the need for better theoretical foundation and the need for higher reporting rates for external validity indicators in research studies.

Part II—ICare Stakeholder Survey: In their introductory paper on the stakeholder survey, Nitsch et al. describe in detail the development of the overall design of the ICare stakeholder survey which followed a mixed-methods approach to collect data from a range of different stakeholders in the healthcare, university and school settings. The next three papers deal with the results of the stakeholder survey in the healthcare, the university and the school setting, respectively. The first of three articles by Kuso et al. presents stakeholders’ perspectives regarding Internet-based interventions to prevent mental health disorders in adults implemented into healthcare systems in Austria, Germany, Switzerland and Spain. Irish et al. describe the results of the stakeholder survey regarding Internet-based interventions to prevent mental health disorders in students implemented in universities in Austria, Germany, UK, Spain, Switzerland and The Netherlands. Zeiler et al. report on the perspectives of key stakeholders in Austria and Spain on Internet-based interventions to prevent mental health problems implemented in schools. The last two articles shift the focus to guided self-help interventions in the context of the treatment of EDs, targeted either to individuals who currently meet diagnostic criteria and are on a waitlist for treatment(to bridge waiting time), or to carers (parents, partners) of patients currently in treatment. The results are presented country-specific. The perspectives of stakeholders’ views on self-help programmes for EDs patients and caregivers implemented in the healthcare system in Germany are presented by Schmidt-Hantke et al. and for the UK by Yim et al.

To the best of our knowledge, this was the first comprehensive stakeholder survey focusing on Internet-based mental illness prevention which was conducted concurrently in several countries and various settings. Overall, 36 focus groups with representatives from the target groups of ICare interventions and 75 interviews with ED patients and carers and policymakers across the three settings (healthcare, schools, universities) and participating countries were conducted. Furthermore, 423 potential facilitators/enablers filled in an online questionnaire. The findings are in accordance with the existing literature regarding barriers and facilitators for dissemination and implementation of Internet-based treatment in the healthcare system^10–13^ and of face-to-face prevention in the field of mental health in other public health settings like schools.^14–17^

Following the outbreak of the Covid-19 pandemic, a significant increase in the prevalence of mental health disorders is to be expected,^18^^,^^19^ consequently raising the demand for mental health interventions globally. Internet-based interventions are feasible and effective adjuncts to face-to-face interventions in order to deal with the increased need for mental health promotion and treatment. Therefore, Internet-based interventions should be integrated as part of routine care in European healthcare systems,^18^ and their integration should be advanced in other settings like schools and universities, because the associated burden with the Covid-19 pandemic and lockdowns are particularly stressful for younger age groups.^19^ In Germany, e.g. the new Digital Healthcare Act already facilitates access to and reimbursement of digital applications for individuals with mental health problems.^20^ We expect the results of the stakeholder survey and the evidence presented in this Supplement to help facilitate a more rapid dissemination and implementation of the currently evaluated and future online interventions also into different settings in Europe.

## Funding

This project has received funding from the European Union’s Horizon 2020 research and innovation programme under grant agreement No. 634757.

*Conflicts of interest*: None declared.
